# Functional complementation of RNA interference mutants in trypanosomes

**DOI:** 10.1186/1472-6750-5-6

**Published:** 2005-02-09

**Authors:** Filippo Rusconi, Mickaël Durand-Dubief, Philippe Bastin

**Affiliations:** 1 UMR5153 CNRS, USM0503 MNHN, U565 INSERM – 57, rue Cuvier – B.P. 26 – F-75231 – Paris Cedex 05 – France

## Abstract

**Background:**

In many eukaryotic cells, double-stranded RNA (dsRNA) triggers RNA interference (RNAi), the specific degradation of RNA of homologous sequence. RNAi is now a major tool for reverse-genetics projects, including large-scale high-throughput screens. Recent reports have questioned the specificity of RNAi, raising problems in interpretation of RNAi-based experiments.

**Results:**

Using the protozoan *Trypanosoma brucei *as a model, we designed a functional complementation assay to ascertain that phenotypic effect(s) observed upon RNAi were due to specific silencing of the targeted gene. This was applied to a cytoskeletal gene encoding the paraflagellar rod protein 2 (*TbPFR2*), whose product is essential for flagellar motility. We demonstrate the complementation of *TbPFR2*, silenced *via *dsRNA targeting its UTRs, through the expression of a tagged RNAi-resistant *TbPFR2 *encoding a protein that could be immunolocalized in the flagellum. Next, we performed a functional complementation of *TbPFR2*, silenced *via *dsRNA targeting its coding sequence, through heterologous expression of the *TbPFR2 *orthologue gene from *Trypanosoma cruzi*: the flagellum regained its motility.

**Conclusions:**

This work shows that functional complementation experiments can be readily performed in order to ascertain that phenotypic effects observed upon RNAi experiments are indeed due to the specific silencing of the targetted gene. Further, the results described here are of particular interest when reverse genetics studies cannot be easily achieved in organisms not amenable to RNAi. In addition, our strategy should constitute a firm basis to elaborate functional-dissection studies of genes from other organisms.

## Background

RNA interference (RNAi) can be triggered by introduction of long double-stranded RNA molecules (dsRNAs) in cells [1], and proceeds in a number of sequential steps, starting with the cleavage of long dsRNAs into shorter ≈ 21–23 nucleotide-long dsRNAs called short interfering RNAs (siRNAs; these were initially discovered in plants [2]). The enzyme responsible for this chopping (DICER; [3,4]) displays RNase III activity, producing characteristic siRNAs with a phosphorylated 5' end and a two nucleotide-overhanging 3'OH end. These siRNAs enter an RNA-induced silencing complex, or RISC [5,6]. A helicase activity unwinds the two strands of the siRNA, and RISC scans the mRNAs in the cytoplasm and cleaves the molecules that are found complementary to the RISC-contained siRNA [5].

RNA-silencing processes have been described in a variety of organisms: post-transcriptional gene silencing in plants [7,8], quelling in fungi [9], homology-dependent gene silencing in ciliates [10], or RNA interference in worms [1], flies [11,12], trypanosomes [13,14] and mammals [15,16]. It is thought that this machinery has evolved to protect cells against undesirable RNAs, like RNA viruses in plants [17,18], or to limit the mobility of transposable elements in animals [19-21].

While RNAi and associated phenomena constitute exceptional recent basic science findings, they also provided a basis for the elaboration of powerful research tools. RNAi methodologies have been set up to perform reverse-genetics studies in a number of organisms. RNAi potency and flexibility have allowed to perform high-throughput genetic screens in several organisms [22-26]. In mammalian cells, the presence of long dsRNA (*>*50 base pairs) triggers the activation of sequence-unspecific interferon-related pathways [27-29]. To circumvent this difficulty, researchers resorted to the transfection of small interfering RNAs [16] or *in vivo *synthesis of small hairpin RNAs, which were demonstrated to produce gene-specific silencing [27,30,31]; reviewed in [32,33].

However, an siRNA might trigger a number of potential unspecific events such as the degradation of partially complementary mRNA due to cross-hybridization, leading to unspecific RNAi, or the translational arrest due to a micro RNA-like effect where an siRNA hybridizes to a mRNA with one or few mismatches. It is thus of paramount importance to ensure that the phenotypic effects observed as a result of siRNA presence in cells are due to silencing of the target gene only. Two large-scale studies show that siRNA-induced gene silencing of transiently- or stably-expressed mRNA is highly gene-specific and does not produce secondary effects detectable by genome-wide expression profiling [34,35]. In contrast, other works provided evidence that siRNAs can be target-unspecific, with the observation of silencing of genes that had limited sequence homology with the siRNA [36,37]. These reports should prompt scientists to assess the specificity of RNAi-silencing in any experiment. A solution to that problem, that we devised in trypanosomes and which is described in this report, is based on the rescueing of the RNAi-mediated loss-of-function phenotype by expressing an RNAi-resistant version of the target gene.

Trypanosomes are protozoan parasites belonging to the Kinetoplastida order. These unicellular flagellated organisms diverged very early in eukaryotic evolution, and exhibit a number of original features [38-40]. Trypanosomes were amongst the first organisms where RNAi was discovered [13,14], and a number of strategies have been devised to either transiently or permanently induce gene-specific RNAi-silencing in these cells [14,41-43]. Examples of successful RNAi in trypanosomes used flagellar genes as targets which yielded easily monitored phenotypes [44]. From a structural point of view, the most conserved morphological feature of eukaryotic flagella is the axoneme, which is made of nine doublets of outer microtubules plus 2 central microtubules (so-called 9+2 axonemal structure). In trypanosomes, the flagellum not only has that axone-mal structure, but it also has a lattice-like structure called the paraflagellar rod (PFR) that is positioned along the axoneme. The two main components of the PFR are TbPFR2 and TbPFR1, that share 60% primary sequence identity [45,46]. *TbPFR2 *silencing leads to flagellar paralysis and trypanosomes do not swim anymore [13,47]. During the cell cycle, the cell first replicates its mitochondrial DNA (kinetoplast) and starts to grow a new flagellum whilst maintaining the old flagellum in place. Hence, a trypanosome which has two kinetoplasts and two nuclei will be close to completion of its cell cycle and will possess an old and a new flagellum [48]. This aspect is an interesting feature for RNAi-based studies of flagellar morphogenesis, because bi-flagellated cells have an "internal control" flagellum (the old one), while the new one has a phenotype corresponding to the RNAi-based gene knock-down. The presence of both the old and the new flagella in the same cell gives an indication of the time course of events when RNAi is induced in trypanosomes, leading to the appearance of a visible phenotype in the new flagellum while the older one is unchanged because it is not affected by gene silencing.

We previously established the degree of identity between the gene sequences capable of leading to cross-RNAi [47,49]. However, as mentioned earlier, each time a phenotype is observed in RNAi experiments, it is necessary to ensure that it is indeed due to the specific silencing of the targeted gene(s). Inspired by the procedure with which gene knock-out is usually performed (the control experiment is done by re-introducing the knocked-out gene to ensure that the lost function gets complemented), we devised a functional complementation strategy aimed at assessing that RNAi indeed targets the intended gene. This strategy, elaborated using the *TbPFR2 *gene as a model system, involved the silencing of the *TbPFR2 *target *via *its UTRs and the expression of a RNAi-resistant copy of the targetted gene. The RNAi-resistant gene was either a copy of *TbPFR2 *with different UTRs or its *Trypanosoma cruzi *orthologue: *TcPFR2*. We found that inter-species complementation experiments were straight forward. This strategy opens a venue for functional gene dissection experiments where modified gene sequences can be tested for their ability to encode functional protein that can complement the RNAi-based loss-of-function phenotype.

## Results and discussion

### Multiple RNAi on trypanosomes

We wanted to establish if the co-transfection of two distinct dsRNAs, targeting two different genes, could trigger their simultaneous silencing. The genes selected were *TbPFR2 *and *FLA1*; *TbPFR2 *encodes one of the two major components of the paraflagellar rod and is necessary for flagellum motility [13]; *FLA1 *encodes a protein required for flagellum attachment to the cell body [50]. These dsRNAs were transfected simultaneously in wild-type trypanosomes. As a control experiment, we used *GFP *dsRNA.

Cells were monitored for their acquired phenotype 15 h and 22 h after transfection (Table 1). The extinction of TbPFR2 was followed by immunofluorescence microscopy using the L8C4 anti-TbPFR2 monoclonal antibody. *FLA1 *gene silencing was analyzed by differential interference contrast microscopy, as it results in the visible detachement of the flagellum from the cell body (Figure 2).

The transfection of *TbPFR2 *dsRNA yielded potent silencing, as more than 60 % of the cells showed no staining for L8C4 15 h later. Since old flagella pre-exist in cells which were affected by RNAi at the beginning of cell replication, the real percentage of silenced cells is probably higher than 60 %, which is confirmed by the fact that it built up to more than 74 % at time point 22 h. The transfection of *FLA1 *dsRNA produced a phenotype in which the flagellum was detached from the cell body in more than 50 % of the cells. When both dsRNAs were co-transfected, both phenotypes were indeed observed, with similar frequencies to experiments where only one dsRNA was transfected. All the transfected cell populations did show a comparable growth rate (data not shown). The trypanosomes shown in Figure 2 had been transfected with both dsRNAs and the cell on the right is starting cytokinesis. The old flagellum of that cell is detached, while the new flagellum is attached along the cell body. The new flagellum exhibits a dilation of its distal tip, probably corresponding to the accumulation of TbPFR1, that is not assembled but still transported to the distal tip of the flagellum in the absence of TbPFR2 [13]. This observation demonstrates the usefulness of double-transfection experiments also for kinetics analysis. In our case, 22 h after dsRNAs transient transfection, the phenotype due to the *FLA1 *silencing is no longer visible in the new flagellum while that same flagellum still exhibits the phenotype due to the *TbPFR2 *silencing, clearly indicating different turn-over for TbPFR2 and FLA1 proteins.

The RNAi machinery could cope with two different dsRNA populations, without – in our conditions – any visible saturation effect. These results show the feasibility of experiments involving the use of multiple dsRNAs, thus allowing studies on complex processes in the cell physiology. However, such complex experiments can only be envisaged after ensuring that the phenotypes resulting from RNAi are specifically due to silencing of the target gene. In order to address that specific problem, we elaborated a method that involves RNAi experiments on trypanosomes that were engineered to possess an extra RNAi-resistant copy of the targeted gene, leading to functional complementation.

### Gene silencing by dsRNA targeting UTRs

**Figure 1:**
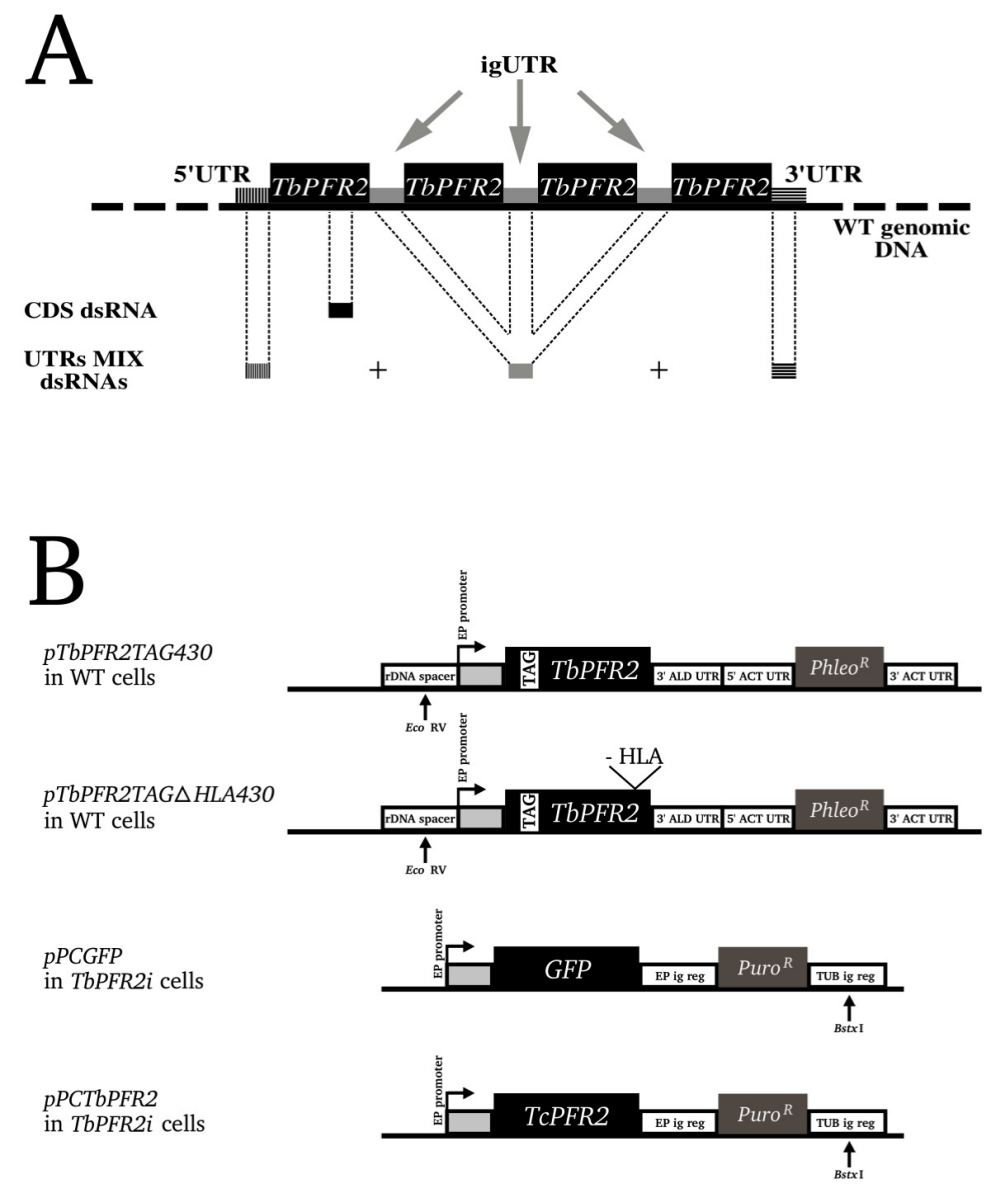
**dsRNAs and plasmids used for transfections**. (A) Not-to-scale schematic representation of the endogenous *TbPFR2 *locus, with four copies of *TbPFR2 *coding sequence and specific UTRs. Regions targeted by RNAi are highlighted. The *TbPFR2 *coding sequence was targeted using CDS dsRNA and the UTRs were targeted all together with a set of dsRNA homologous to the 3' UTR, the intergenic UTR (igUTR) and the 5' UTR (UTRs MIX dsRNAs). (B) Not-to-scale representation of the constructs used for the transfection of WT cells (pTbPFR2TAG430 and pTbPFR2TAG430-ΔHLA; integration in the rDNA spacer) or *TbPFR2i *cells (pPCGFP and pPCTcPFR2; integration in the tubulin intergenic region). Large boxes represent protein coding sequences (black boxes: proteins of interest; grey boxes: antibiotic-resistance activities). Each plasmid was linearized with the indicated restriction enzyme prior to transfection into the cells. *3' ALD UTR*: 3' UTR of the aldolase gene; *ACT UTR*: 5' or 3' UTR of the actin gene; *EP ig reg*: EP procyclin intergenic region; *TUB ig reg*: tubulin intergenic region.

As a model for this study, we chose the *TbPFR2 *gene, which is present in four copies in the WT trypanosome genome (Figure 1A), all transcribed as a single long polycistronic mRNA. All these gene copies are separated by three identical intergenic UTRs (igUTR), while the first copy has a unique 5' UTR and the last copy has a unique 3'UTR. Three types of dsRNA populations were used in our experiments, and termed as follows. dsRNA homologous to the *GFP *sequence was labelled "GFP dsRNA"; dsRNA homologous to coding sequence of the *TbPFR2 *gene was labelled "CDS dsRNA"; finally, the mixture of three dsRNAs homologous to the 5' UTR, igUTR and 3'UTR of the *TbPFR2 *gene was termed "UTRs MIX dsRNAs" (Figure 1A). These dsRNAs were transfected into three cell lines: WT, *TbPFR2tag *and *TbPFR2tag-ΔHLA *(see Methods). For each experiment, the presence or absence of TbPFR2 in the new flagellum of bi-nucleated/bi-flagellated cells was monitored by immunofluorescence 14 h after the transfection (Table 2).

Reports in [14,24] showed that RNAi silencing of a gene can be accomplished by targeting transcribed non-coding sequences. Here, we wanted to make sure that this kind of experiment was still feasible with a more complex system such as the *TbPFR2 *multigene locus, where multiple and distinct UTRs regulate the expression of four *TbPFR2 *isogenes. We first transfected WT trypanosomes with GFP dsRNA as a negative control and did not detect any *TbPFR2 *silencing (Figure 3A). Second, WT trypanosomes were transfected with the CDS dsRNA: 88 % of cells showed typical *TbPFR2 *silencing with an anti-TbPFR2 immunofluorescence showing that the protein was missing from the new flagellum (Figure 3B). Finally, the WT trypanosomes were transfected with the UTRs MIX dsRNAs, yielding the same phenotype as for the CDS dsRNA, although the silencing appeared less pronounced (Figure 3C and Table 2). Overall, these results demonstrate that RNAi could efficiently silence all of the *TbPFR2 *gene copies by targeting non-coding sequences present at the mRNA level.

When WT trypanosomes were transfected with *TbPFR2 *dsRNA complementary to only one UTR, the cells did not display any specific phenotype (data not shown). This observation is probably explained by the organization of the *TbPFR2 *locus: the polycistronic transcript is rapidly spliced into three different types of mRNA, each encoding one of the four copies of *TbPFR2 *[51-53]. Thus, even if one type of *TbPFR2 *RNA is destroyed, the three remaining ones would likely provide enough RNA to synthesize TbPFR2 levels compatible with normal PFR formation.

To demonstrate that the silencing observed upon transfection of WT trypanosomes with the UTRs MIX dsRNAs was due to the actual targeting of *TbPFR2*, we used two cell lines expressing a supplementary tagged *TbPFR2 *gene copy. The *TbPFR2tag *cell line expresses the TbPFR2-TAG protein which correctly localizes to the flagellum.

The *TbPFR2tag-ΔHLA *cell line expresses TbPFR2-TAG-ΔHLA, lacking the HLA tripeptide, which prevents its localization to the flagellum. To determine both the cellular localization of the tagged TbPFR2 proteins (TbPFR2-TAG and TbPFR2-TAG-ΔHLA) and the completeness of the PFR assembly, immunofluorescence experiments were carried out with the BB2 and ROD-1 antibodies; the former recognizes the Ty-1 epitope tag present on the two tagged TbPFR2 proteins [54], while the latter is a marker for full PFR assembly [55,56].

GFP dsRNA was transfected as a negative control in each cell line. As expected, this did not yield any *TbPFR2 *silencing: TbPFR2 was decorated in both the old and new flagella by the anti-TbPFR2 antibody, and the PFR could be assembled fully, as evidenced by its staining with the ROD-1 antibody (data not shown, Table 2). In *TbPFR2tag *cells, TbPFR2-TAG was able to localize to the PFR, as evidenced by the PFR decoration with BB2 (red color, Figure 4A). In contrast, TbPFR2-TAG-ΔHLA failed to do so in *TbPFR2tag-ΔHLA *cells, and the BB2 signal was detected in the cytoplasm, as expected (red color, Figure 4D).

We next compared *TbPFR2tag *trypanosomes after transfection with either CDS dsRNA or UTRs MIX dsRNAs. *TbPFR2tag *cells transfected with CDS dsRNA had a flagellum not (or faintly) decorated with the anti-TbPFR2 antibody, demonstrating that both the WT and the recombinant *TbPFR2 *gene copies were effciently silenced (Table 2). That result was confirmed with anti-TAG immunofluorescence that showed no staining of the flagellum, demonstrating that TbPFR2-TAG was absent (no red color, Figure 4B). This lack of both TbPFR2 and TbPFR2-TAG led to an incomplete assembly of the PFR, which was therefore not decorated with the ROD-1 antibody (no yellow color, Figure 4B). In contrast, cells transfected with the UTRs MIX dsRNAs exhibited a WT phenotype, with only 2 % of the cells displaying *TbPFR2 *silencing in the flagellum (Table 2). In this case, the tagged protein was expressed, leading to complete assembly of the PFR (yellow color, Figure 4C) because the protein is functional and localized to the flagellum (red color, Figure 4C; [56]). This remarkable result indicates a complementation phenomenon that is explained by the fact that the tagged *TbPFR2 *gene was not silenced, as it was expressed from a coding sequence flanked by UTRs from the expression vector: from the 5' UTR of the procyclin gene and from the 3'UTR of the aldolase gene (Figure 1B; [57]).

To definitely demonstrate that the complementation described above is indeed due to the expression of functional TbPFR2-TAG, we transfected the same dsRNA into *TbPFR2tag-ΔHLA *trypanosomes expressing a modified TbPFR2 protein missing three amino acids (that is nine nucleotides out of 1800). TbPFR2tag-ΔHLA does not access the flagellar compartment and thus cannot be functional [58]. Transfecting either CDS dsRNA or UTRs MIX dsRNAs produced cells in which the new flagellum was not (or faintly) decorated by the anti-TbPFR2 antibody (Table 2). TbPFR2-TAG-ΔHLA was not decorated by the anti-TAG antibody when cells were transfected with CDS dsRNA (red color, Figure 4E), indicating that both the WT and the tagged *TbPFR2 *copies were silenced, thus leading to an incomplete PFR edification (yellow color, Figure 4E). In contrast, transfection of UTRs MIX dsRNAs did not prevent the expression of the recombinant TbPFR2-TAG-ΔHLA protein, as it appeared stained by the anti-TAG antibody (red color, Figure 4F). However, that non-functional protein could not participate in the construction of the PFR, as shown by the absence of ROD-1 signal in the new flagellum, since it cannot access the flagellum (yellow color, Figure 4F).

RNA-directed RNA polymerase activity (RdRP) has been implicated as one possible step in the formation of siRNA in fungi [59], plants [17,18], and worms [60]. The fact that we could specifically silence WT *TbPFR2 *by targeting its UTRs, without interfering with the tagged *TbPFR2 *genes, suggests that spreading of silencing beyond the initial targeted sequence does not occur in trypanosomes [61-64].

### Functional complementation with orthologue genes

We next asked if an RNAi-mediated loss of function could be complemented by the expression of a gene orthologue to the silenced one. The system used to answer that question involved the *TbPFR2i *cell line – that expresses *TbPFR2 *dsRNA under the control of a tetracycline-inducible promoter [47] – into which constitutive expression of *Trypanosoma cruzi TbPFR2 *orthologue (*TcPFR2*) was established using stable transfection procedures. TbPFR2 and TcPFR2 proteins share 90 % identity (both of them are recognized by the anti-TbPFR2 L8C4 antibody), but their gene sequences have diverged enough for us to envisage that the RNAi-silencing of *TbPFR2 *would not affect significantly the introduced *TcPFR2 *gene (83 % overall nucleotide identity). We thus created two new cell lines based on the previously described *TbPFR2i *cells [47] (see Methods). TbPFR2 expression and cell motility were analyzed.

Our first experiment showed that the *PCGFP *cells constitutively expressed GFP, as detected by microscope observation of living cells (data not shown). Both the *PCGFP *and *PCTcPFR2 *cell lines were induced to express *TbPFR2 *dsRNA for 48 hours. Immunofluorescence revealed that non-induced *PCGFP *cells exhibit a WT-like phenotype (Figure 5A, *-*TET). When these cells were induced with tetracycline, expected *TbPFR2 *silencing occurred (Figure 5A, +TET). Non-induced *PCTcPFR2 *cells displayed an intense anti-TbPFR2 antibody decoration with bright dots in the cytoplasm, indicative of TcPFR2 overexpression (such overexpression by the EP procyclin promoter is frequent; Figure 5B, *-*TET). When these same cells were tetracycline-induced, the flagellar staining was still perfectly visible, at a level comparable to the one previously observed in the non-induced *PCGFP *control cells (Figure 5B, +TET). Bright dots previously observed had disappeared, probably as a result of *TbPFR2 *silencing. The fact that the paraflagellar rod was still neatly decorated by the anti-TbPFR2 antibody demonstrated that the *structural *inter-species complementation had indeed taken place in these cells, with TcPFR2 being effectively located at the flagellum.

Did these structurally-complemented cells show a *functional *complementation, i.e. a normal flagellum motility (hence a normal cellular mobility)? To address this question, we performed a sedimentation assay [56] on non-induced and tetracycline-induced *PCGFP *and *PCTcPFR2 *trypanosomes (Figure 6). Non-induced *PCGFP *cells showed a little tendency to sediment due to the fact that expression of *TbPFR2 *dsRNA in *TbPFR2i *cells is partially leaky, producing low amounts of *TbPFR2 *dsRNA even in the absence of tetracycline ([43]; Durand-Dubief and Bastin, unpublished data). When expression of *TbPFR2 *dsRNA was fully induced, motility stopped leading to increased sedimentation (Figure 6, left panel). In contrast, expression of *TbPFR2 *dsRNA in *PCTcPFR2 *cells did not reduce motility (Figure 6, right panel). That result definitely demonstrates that the ortholog protein TcPFR2 fully complemented the loss of function resulting from *TbPFR2 *silencing.

The complementation described above shows the robustness of our strategy, because TbPFR2 and TcPFR2 are highly similar (they share 82 % identity at the nucleotide level [65]) and are nonetheless correctly differenciated by the RNAi machinery. However, our complementation strategy might be more diffcult to implement when the gene studied is too similar to the *T. brucei *counterpart. While this unfavorable case might happen with extremely evolutionarily-related organisms, studies have shown that the overall genetic sequence identity between *Trypanosoma brucei *and *Trypanosoma cruzi*, for example (the closest evolutionarily-related organisms envisaged for these studies), is roughly 80 % ([65] and [66]). [47] showed that this identity percentage is still compatible with an RNAi-based complementation strategy. It goes without saying that when the organisms are evolutionarily-distant, gene sequences diverge more rapidly than the protein sequences, thus laying off a field where our strategy can be implemented with good confidence that complementation will occur.

## Conclusions

In this report, we demonstrated that RNAi-mediated silencing of a gene by targeting its UTRs is useful in studies where the loss of function resulting from this silencing must be complemented with the expression of an RNAi-resistant copy of the silenced gene, in order to demonstrate that the phenotype is indeed due to silencing of that gene, and not to inactivation of another one. The results obtained in this work are of particular interest when reverse-genetics studies cannot be easily achieved in organisms not amenable to RNAi, like *Leishmania *[67] or *Trypanosoma cruzi *[68], or where genetics experiments are hardly set up, like mammals. When genes from these organisms are to be studied, a complementation experiment can be set up as a three-step procedure whereby: 1) the ortholog gene in *Trypanosoma brucei *is RNAi-silenced and the loss-of-function phenotype is established; 2) *T. brucei *cells are engineered to ensure constitutive heterologous expression of the gene of interest, still allowing RNAi-mediated silencing of the *T. brucei *gene; 3) function of the investigated gene is assessed by checking if the loss-of-function phenotype observed in the first place gets complemented. Additionally, one application of the strategy described herein is genetic functional dissection, which is of interest when protein domains are to be characterized with respect to their function (e.g. the HLA tripeptide sequence in TbPFR2 that localizes the protein to the flagellum).

Complementation had previously been demonstrated following transformation of mammalian cells with EGFP siRNA and expression of a codon-modified, but functional, EGFP version [69]. Our strategies are increasing flexibility for complementation studies after RNAi as unmodified genes can be used for rescue.

## Methods

### Trypanosomes

The procyclic *T. brucei brucei *strain 427 (or its derivatives) was used throughout this work. Cells were cultured at 27°C in semi-defined medium 79 (SDM 79) containing 10% foetal calf serum. *PFRAi *cells were described in [47]. The *TbPFR2i *trypanosomes can be tetracycline-induced to express *TbPFR2 *dsRNA, thus eliciting an RNAi response against that gene. Note that this cell line is referred to as *TbPFR2i *in this article because of a change in the gene nomenclature [70].

### RNAi assays by transient transfection

RNA was synthesized *in vitro *with T3 and Sp6 polymerases using PCR products as templates [71]. The following primers (incorporating T3 or Sp6 promoters) were used:

for *GFP *(from the nucleotide coding sequence 476–691 of the *EGFPN2 *gene; Clontech), *AATTAACCCTCACTAAAGGGAGAAG *AACGGCATCAAGGTGAAC (T3 promoter italicized) and *ATTTAGGTGACACTATAGAAG *AGTGATCCCGGCGGCGGTCACG (Sp6 promoter italicized);

for *FLA1*, *AATTAACCCTCACTAAAGGGAGA *CCAAACCGTGGGCACCAAGG (T3 promoter italicized) and *ATTTAGGTGAACTATAGAAGAG *GTGGGATGATTAAAACGAGC (Sp6 promoter italicized);

for the *TbPFR2 *5' untranslated region (5' UTR; nucleotide sequence [-545→-1] upstream of *TbPFR2 *ATG start codon), *AATTAACCCTCACTAAAGGGAGA *(T3 promoter) and *ATTTAGGTGACACT-ATAGAAGAG *(Sp6 promoter);

for the *TbPFR2 *intergenic untranslated region (igUTR), *AATTAACCCTCACTAAAGGGAGA *CGCTGCGCTTAAATGTCTT (T3 promoter italicized) and *ATTTAGGTGACACTATAGAAGA *GTGATGCTTTATTGCTTTCT (Sp6 promoter italicized);

for the *TbPFR2 *3'untranslated region (3'UTR; nucleotide sequence [1→533] downstream of the *TbPFR2 *TAG stop codon), *AATTAACCCTCACTAAAGGGAGA *(universal T3 promoter) and *ATTTAGGTGACACTATAGAAGAG *(universal Sp6 promoter);

for the *TbPFR2 *coding sequence (CDS; nucleotide coding sequence [1084→1358]), *ATTTAGGTGACACTATAGA *GAGGTGAAGCGCCGTATTGAGGA (Sp6 promoter italicized) and *AATTAACCCTCACTAAAGGGAGA *GTTTTGTACAGGCGACGGAA (T3 promoter italicized);

Figure 1A shows the *TbPFR2 *locus and the position of the two dsRNA populations that were used, and their homology to either the coding sequence (labelled "CDS dsRNA") or the different 5'UTR, igUTR and 3'UTR all together (labelled "UTRs MIX dsRNAs"). A third dsRNA, homologous to the *GFP *gene is labelled "GFP dsRNA" throughout this work and was used as a control dsRNA. dsRNA was introduced into trypanosomes by electroporation, as described [14].

### Plasmids

Plasmid pPC was generated from plasmid pSk1-GFP [50] as follows: pSk1-GFP was digested with *Hin*d III and *Eco *RI to remove the *GFP *gene. Oligonucleotides *AGCT *GTCTAGCGATATCGGATCCG (forward) and *AATT *CGGATCCGATATCGCTAGCA (reverse) were annealed (protruding ends italicized) and the resulting double-strand oligonucleotide was ligated into the pSk1-GFP plasmid, resulting in the insertion of a poly-linker containing restriction sites *Cla *I, *Hin*d III, *Nhe *I, *Eco *RV, *Bam *HI and *Eco *RI (Branche and Bastin; unpublished data). Plas-mid pPCTcPFR2 was generated as follows: amplification of the *TcPFR2 *gene was performed using *Trypanosoma cruzi *genomic DNA (kind gift of Cécile Gallet and Philippe Grellier, MNHN) and the two primers TcPFR2H (GAGTCTAAGCTTATGAGCTACAAGGAGGCATC) and TcPFR2ER (GCGTGGAATTCTTACTGTGTGATCTGCTGCAC). Both the amplified DNA fragment and the pPC plasmid were digested with *Eco *RI and *Hin*d III. The fragment was ligated into pPC so as to yield the plasmid pPCTcPFR2 (Figure 1B).

### Cell lines

The different constructs used to transform trypanosomes are shown on Figure 1B. The cell lines were established as follows.

#### WT-derived trypanosomes constitutively expressing TbPFR2-TAG proteins

The *TbPFR2tag *cell line was derived from the WT cell line into which the pTbPFR2TAG430 plasmid [72] was transfected. The recombinant cells constitutively expressed the TbPFR2-TAG protein, that is localized in the flagellum (Fig 4D). Tagged TbPFR2 is known to be functional [56,72]. In contrast, transformation of WT cells with the pTbPFR2TAGΔHLA430 plasmid lead to the expression of slightly modified TbPFR2 protein, missing only three amino acids, that failed to enter the flagellum compartment and hence was found in the cell body cytoplasm [58] (Fig 4G). This cell line was called *TbPFR2tag-ΔHLA*. After electroporation [73], cells were grown overnight and then distributed in 24-well plates in the presence of phleomycin (2 *μ*g/mL) for selection.

#### TbPFR2i-derived trypanosomes constitutively expressing GFP and TcPFR2

*TbPFR2i *cells [47] constituted the genetic background into which we established the *PCGFP *and *PCTcPFR2 *new cell lines. The *PCGFP *cell line was established by transfecting *TbPFR2i *cells with plasmid pPCGFP after linearization with *BstX *I. For establishing the *PCTcPFR2 *cell line, the pPCTcPFR2 plasmid was linearized with *BstX *I and transfected into *TbPFR2i *cells. Recombinant cells were selected by addition of puromycin (1 *μ*g/mL), phleomycin (2 *μ*g/mL), G418 (15 *μ*g/mL) and hygromycin (20 *μ*g/mL) to the culture medium.

### Immunofluorescence and microscopy

Three different monoclonal antibodies were used as hybridoma supernatants: L8C4, IgG recognizing *T. brucei *TbPFR2 and cross-reacting with *T. cruzi *orthologue TcPFR2 [74]; BB2, IgG recognizing the Ty-1 tag of the TbPFR2-TAG and TbPFR2-TAG-ΔHLA recombinant proteins [54]; and ROD-1, IgM recognizing a doublet of minor PFR proteins [55]. For immunofluorescence, trypanosomes were spread onto poly-L-lysine-coated slides, fixed in cold methanol and processed as described [75]. Experiments involving the use of L8C4 only were performed with an FITC-conjugated anti-mouse IgG secondary antibody. Double-staining experiments using BB2 and ROD-1 were performed with a TRITC-conjugated specific anti-mouse IgG secondary antibody and an FITC-conjugated specific anti-mouse IgM secondary antibody. DNA was systematically stained with 4',6-diamidino-2-phenylindole (DAPI). Slides were examined with a Leica DMR microscope, images were captured using a cooled CCD camera (Cool Snap HQ, Roper Scientific) and processed with the GNU image manipulation program version 2 [76].

### Cell sedimentation assay

The trypanosome sedimentation assay was performed as described in [56]. Briefly: trypanosomes were grown at ≈ 5.10^6 ^cells/mL in normal culture medium, with or without 48 hour tetracycline induction. 1 mL of these cultures was dispensed to 5 plastic spectrophotometry cuvettes, for time points 0, 2, 4, 6, 8 hours, and left still. At each time point, the optical density at 600 nm was measured twice: first without mixing (O.D._no mix_) and second after mixing the cuvette (O.D._mix_). Data were plotted as a function of time.

## Authors' contributions

**Figure 2:**
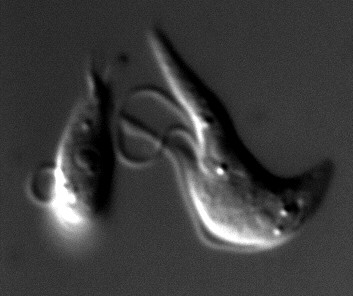
**Silencing of FLA1 and TbPFR2 in Trypanosoma brucei**. Wild type cells were simultaneously transfected with *FLA1 *and *TbPFR2 *dsRNA. At 22 h post-transfection, live cells were observed by differential interference contrast microscopy. Note the detached old flagellum and the dilation at the tip of the new flagellum (shorter one) of the rightmost cell.

**Figure 3:**
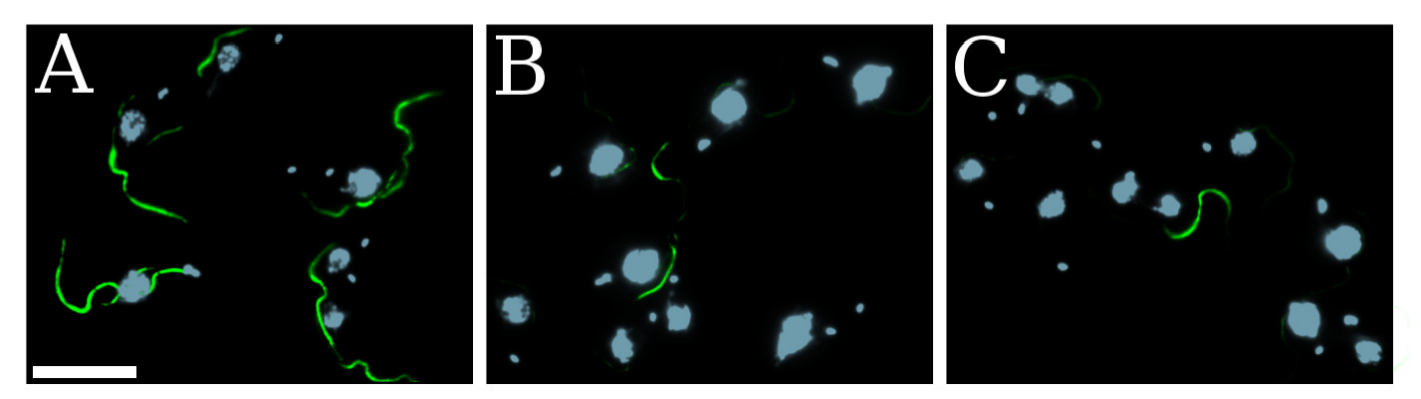
**TbPFR2 silencing by targeting its UTRs all together**. WT cells were transfected with GFP dsRNA (A), CDS dsRNA (B) or UTRs MIX dsRNAs (C). At 14 h post-transfection, cells were treated for immunofluorescence using the L8C4 anti-TbPFR2 antibody (green) and counterstained with DAPI (blue). Cells of interest are bi-nucleated/bi-flagellated. Transfection of GFP dsRNA did not produce any specific phenotype. Both the CDS dsRNA and the UTRs MIX dsRNAs successfully silenced *TbPFR2*. Scale bar: 10 *μ*m. See text for details.

**Figure 4:**
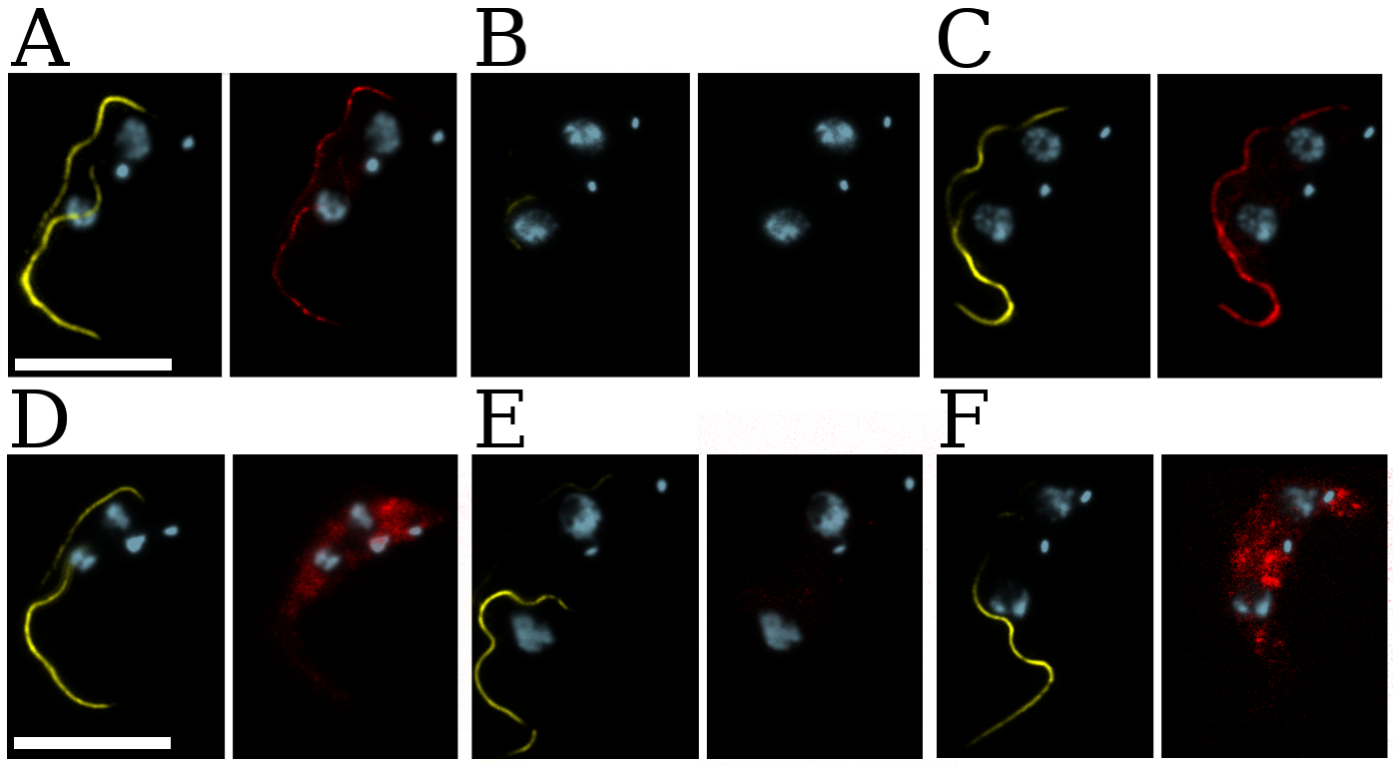
**Structural complementation of RNAi mutants**. *TbPFR2tag *(panels A–C) or *TbPFR2tag-ΔHLA *(panels D–F) cells were transfected with GFP dsRNA (first column), CDS dsRNA (second column) or UTRs MIX dsRNAs (last column). At 14 h post-transfection, cells were treated for immunofluorescence with both the ROD-1 antibody (marker for full PFR assembly, yellow) and the BB2 anti-TAG antibody (red). All cells were counterstained with DAPI (blue). Cells of interest are bi-nucleated/bi-flagellated. Transfection of GFP dsRNA (A, D) did not produce any specific phenotype, whatever the recipient cells: the paraflagellar rod could assemble normally (yellow). As expected, TbPFR2-TAG localized to the flagellum (A, red), while TbPFR2-TAG-ΔHLA accumulated in the cytosol (D, red). The CDS dsRNA (B, E) successfully silenced the WT and the tagged *TbPFR2 *genes on both cell lines: no tagged TbPFR2 was stained with BB2; the paraflagellar rod could not assemble (no yellow signal is visible in the new flagellum). When using the UTRs MIX dsRNAs in *TbPFR2tag *and *TbPFR2tag-ΔHLA *cells, the wild-type *TbPFR2 *gene is silenced (see Figure 3). However, only in the *TbPFR2tag *cells does TbPFR2-TAG complement the missing TbPFR2 protein: TbPFR2-TAG was stained in the flagellum in (C, red). TbPFR2-TAG-ΔHLA failed to complement in the *TbPFR2tag-ΔHLA *cells: TbPFR2-TAG-ΔHLA was stained in the cytoplasm in (F, red). Thus, the paraflagellar rod could assemble fully in (C, yellow) but failed to do so in (F, yellow). Scale bar: 10 *μ*m.

**Figure 5:**
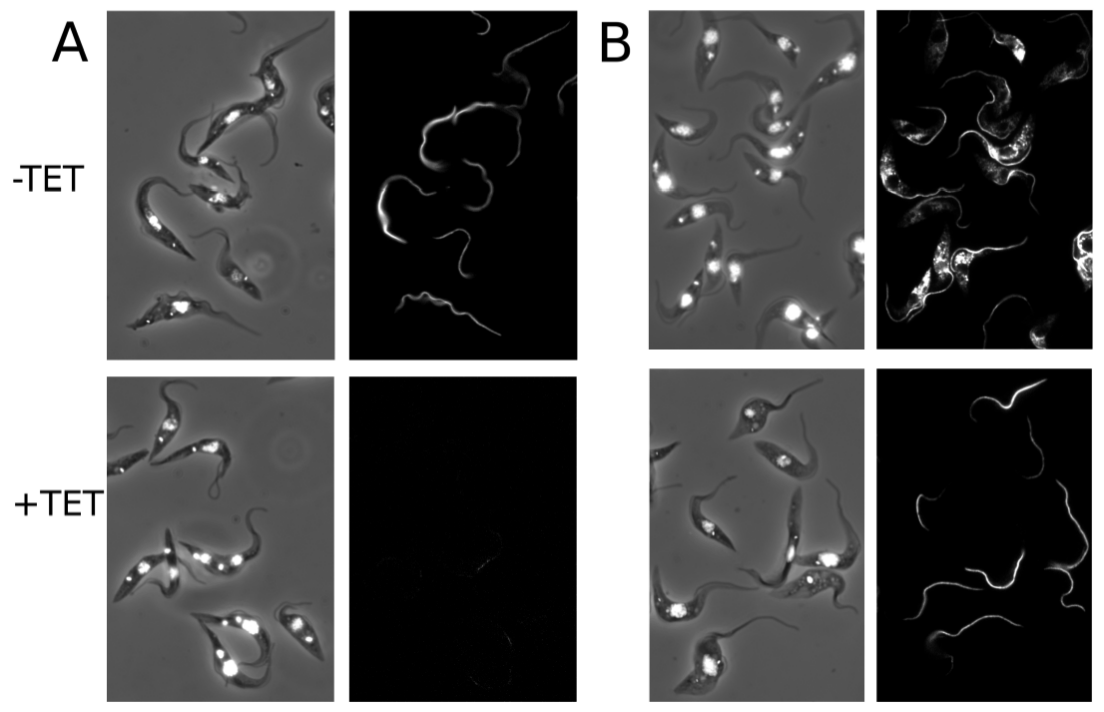
**Interspecies structural complementation of RNAi mutants**. *TbPFR2i*-derived trypanosomes, constitutively expressing GFP (panel A; *PCGFP *cells) or TcPFR2 (panel B; *PCTcPFR2 *cells) were cultured for 48 h in the absence (-TET) or in the presence (+TET) of tetracycline. In these *TbPFR2i*-derived cells, tetracyline-induction triggers RNAi-silencing of the endogenous *TbPFR2 *gene. The cells were then treated for immunofluorescence using L8C4 as an anti-TbPFR2 (right panel, black background) and counterstained with DAPI (left panel, merged with phase contrast image). Non-induced *PCGFP *cells show normal flagellar staining (A, -TET) while induced cells show an almost total loss of flagellar signal (A, +TET) due to silencing of *TbPFR2*. Non-induced *PCTcPFR2 *cells show an intense L8C4 signal in the flagellum and sometimes in the cytoplasm, due to overexpression (B, -TET). Upon tetracycline-induction of these cells, the flagellar L8C4 decoration did not disappear, indicating that *TcPFR2 *was not subject to RNAi and that TcPFR2 could successfully localize to the flagellum (B, +TET).

**Figure 6:**
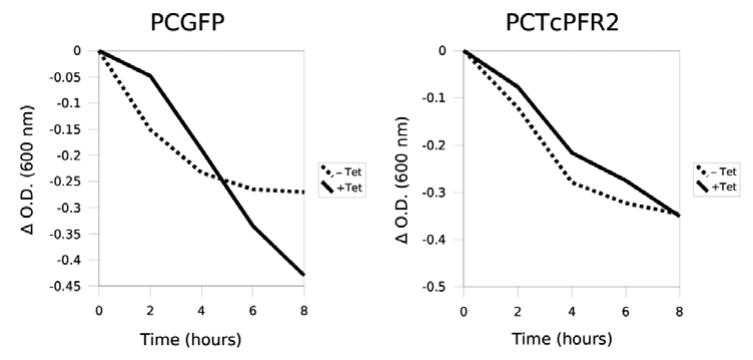
**Phenotype analysis by sedimentation assay**. *PCGFP *and *PCTcPFR2 *trypanosomes were induced (+Tet) with tetracycline for 48 h or non-induced (-Tet) and grown in cuvettes. Non-motile cells do not swim and sediment to the bottom of the cuvette, leading to a reduction in optical density that can be monitored over time. The *PCTcPFR2 *cells show an almost perfect functional complementation phenotype, as induced and non-induced cells had virtually identical sedimentation curves.

F.R. carried out most of the experiments reported and wrote the manuscript, M.D.-D. performed the double transfection reported at Table 1 & Figure 2 and P.B. conceived the study and participated in its design and coordination.

## Figures and Tables

**Table 1:** Silencing effciencies after transfection of various dsRNAs. WT trypanosomes were transfected with *TbPFR2*, *FLA1 *or *GFP *dsRNA.

		Time (hours)		
		
dsRNA	Phenotype (%)	0 h	15 h	22 h
*TbPFR2*	*TbPFR2 *silencing	0	61.2 (n = 1069)	74.2 (n = 1069)
*FLA1*	*FLA1 *silencing	0	56 (n = 586)	54 (n = 472)
*TbPFR2 *+ *GFP*	*TbPFR2 *silencing	0	56.2 (n = 301)	70.9 (n = 243)
*TbPFR2 *+ *FLA1*	*TbPFR2 *silencing	0	53.6 (n = 349)	69.7 (n = 210)
	*FLA1 *silencing	0	59 (n = 349)	53 (n = 210)

**Table 2:** Silencing effciencies after transfection of various dsRNAs.

Cell line	*TbPFR2 *silencing in the new flagellum (% bi-flagellated cells)	
	dsRNA transfected	
	
	*TbPFR2 *CDS	*TbPFR2 *UTRs MIX
WT	88 % (n = 53)	62 % (n = 147)
*TbPFR2tag*	68 % (n = 113)	2 % (n = 51)
*TbPFR2tag-ΔHLA*	78 % (n = 100)	72 % (n = 100)

WT, *TbPFR2tag *and *TbPFR2tag-ΔHLA *trypanosomes were transfected with *GFP*, *TbPFR2 *CDS or *TbPFR2 *UTRs MIX dsRNAs and bi-nucleated/bi-flagellated cells were counted for the status of their new flagellum. The data presented in this table correspond to the immunofluorescence experiment described in Figure 3 (see text for details).
